# Linguistic Traces of Subjectivity and Dissent. A Discursive Analysis of Inclusive Language in Argentina

**DOI:** 10.3389/fsoc.2021.633330

**Published:** 2021-06-16

**Authors:** Carolina Tosi

**Affiliations:** Instituto de Lingüística, Facultad de Filosofía y Letras, Universidad de Buenos Aires, Consejo Nacional de Investigaciones Científicas y Técnicas, Buenos Aires, Argentina

**Keywords:** inclusive language, gender, identity, sexual dissidence, polyphony, argumentation

## Abstract

In Argentina, the so-called “inclusive language” aims at avoiding the bias for a particular sex or gender and objects to a grammatical binary system (feminine – masculine). Although in most Spanish-speaking countries, inclusive language has been limited to the realms of activism, gender studies and a certain type of public administration, in Argentina, its use has been extended to different social spheres, mostly urban. Considering such context, this work aims to investigate the inclusive language in Spanish and characterize its most relevant resources in a series of public texts that circulated in Argentina between 2018 and 2020. On the one hand, its origin is explained, differentiating it from non-sexist language and the different theoretical positions around inclusive language are exposed. On the other hand, from the Dialogical Approach to Argumentation and Polyphony, this paper proposes to address inclusive language resources as subjectivity and polyphony marks which evidence certain aspects of the discourse of patriarchy, with respect to which there is dissent; therefore, inclusive language resources show viewpoints that were once silenced and rejected. For this, a corpus of various speeches is addressed, made up of outdoor urban inscriptions, flyers (advertisements), audiovisual informative speeches and digital press, written in inclusive language, between 2018 and 2020. Throughout the paper it is warns that the inclusive language marks, such as –*e* and *x*, are traces of the “heterogeneity shown marked” that object to grammatical binarism and convey comments by the subject about their own enunciation, alluding to the image of previous sexist and patriarchal discourses with whom they disagree. The analysis reveals that the words or expressions in which inclusive language resources are employed *(-e* and *x*) work as traces of harassed identities and manifest comments by the speaker on their own enunciation. This way, this research shows that gender inclusive language holds conflict linguistic marks which point to historically denied dissidence forms, linked to gender identity and the assertion of collective rights. Finally, this article aims at, on the one hand, contributing to the description of Argentinean Spanish, and on the other, promoting reflection in favor of linguistic education. Undoubtedly, opening instances of debate on the subject can have an impact on the deepening of linguistic reflection and the training of speakers who contribute to forging a more egalitarian society, one which is inclusive and respectful of differences.

## Introduction

For several decades, studies with a gender perspective have questioned the androcentrism and the patriarchal character of language. If we refer to the Spanish language, it has been deemed *sexist*, insofar as it gives men a central position in the world, making women and sexual dissidence invisible. Among the sexist forms attributed to the Spanish language, morphological aspects are usually mentioned, such as the generic or universal masculine gender, and also lexical forms are noted, together with the asymmetrical employment of address forms and the use of the masculine variant for professions and titles.

Then, from the postulate that the use of the masculine to refer to the two sexes does not manage to show the woman, and that this comes from the lack of symbolic representation of women in the language (Alario et al., 1995: 4), various guides–especially since the 90s–proposed alternative ways to tend toward a non-sexist language that evidences women, such as doubling or double mentioning (“chicos and chicas”), and the use of bars (“chico/as”), as will be explained in the work. Later, as of 2000, the so-called “inclusive language” emerged, promoted by studies with a gender perspective, that aims at gender equality and the visibility of dissident identity groups. With the aim of avoiding bias toward a particular sex or gender^1^ and objecting to the binary system of the Spanish language (feminine-masculine), the numerous guides to inclusive language^2^ bring into play different resources, such as the use of generic nouns without determiners, collective nouns, abstract nouns, nominalizations, paraphrases, and graphic resources that are put forth as an alternative to the generic masculine: @, x, *el* ^*^, and *la* –*e*.

It is worth mentioning that, despite the fact that, in most Spanish-speaking countries, inclusive language has been limited to realms of activism, studies with a gender perspective and certain public administration sectors, its use in Argentina has extended to various social areas, especially urban ones. Starting with the activist movements of *Ni Una Menos* (Not a Woman Less) (2015) and those that supported the *Interrupción Voluntaria del Embarazo* (Voluntary Interruption of Pregnancy) Bill (2018), inclusive language broke into the voices of the protesters and made long overlooked identities visible.

Based on previous works (Tosi, 2019, 2020; Sardi and Tosi, 2021), and employing the Dialogical Approach to Argumentation and Polyphony framework (García Negroni, 2018, 2019), the present article addresses inclusive language and characterizes its resources as linguistic marks of conflict that point to historically denied dissidence identities. The hypothesis I present consists in that inclusive language marks, such as –y and x, are traces of the heterogeneity shown marked (Authier-Revuz, 1984) that object to grammatical binarism and convey comments by the subject about their own enunciation, alluding to the image of previous sexist and patriarchal discourses with whom they disagree. The article is structured as follows. First, the methodology and theoretical framework used are explained. Second, studies are presented that examine the impact of language on gender identity, on the one hand, and the sexist uses of Spanish, on the other. Third, the proposal of inclusive language is explored and some theoretical perspectives that interpret it are laid out. Then, the analysis of the corpus is carried out and the results obtained are presented. Finally, the discussion and conclusions are included.

## Background and Method

### Methodology

The design of the research carried out for this analysis was based on qualitative methodology, since qualitative approaches are better suited to investigate “delimited and focused groups and segments of social histories from the perspective of the actors, of relationships, and for the analysis of discourses and documents” (De Souza Minayo, 2009: 47). This type of method allows for the unveiling of social processes related to particular objects of study and motivates the construction of new studies and the creation of concepts and analytical categories. As it is known, the qualitative method is usually employed in the humanities and social sciences, because it is the method that best responds to their objectives and needs, because it is characterized by an empirical and progressive systematization of knowledge, which leads to the understanding of the internal logic of the group or process under study (De Souza Minayo, 2009). Therefore, we have applied a qualitative methodology, since it has allowed us to approach, in a systematic and progressive way, the phenomenon of inclusive language in certain discursive practices that unfold in urban spaces mostly. Regarding the latter aspect, we have verified that inclusive language enjoys wide circulation in Argentinean cities, and therefore it is usually characterized as a predominantly urban phenomenon (Moure, 2018).

A *discursive corpus* (Courtine, 1981) has been employed, made up of discourses that have marks of inclusive language, and which have been generated in the last 2 years (2018–2020) in different cities of the Province of Buenos Aires and the City of Buenos Aires. The decision regarding the temporal segment was made because, in 2018, inclusive language gained notoriety when it was used among the demonstrators who supported the *Interrupción Voluntaria del Embarazo* (Voluntary Interruption of Pregnancy) Bill. Its circulation was commented on in the mass media covering the event, and on many occasions, there were heated debates about the novel linguistic phenomenon. Finally, in order to obtain a representative sample, we extended the corpus collection period until September 2020.

As for the types of discourse that make up the corpus, we include a wide variety of genres, formats and media, ranging from urban outdoor inscriptions (Gándara, 2002), ranging from non-institutionalized practices, that is, those not regulated by an institution, made in some cities of Buenos Aires, by subjects anonymously and even texts that present a controlled writing and correction process, such as texts circulating on social networks and the virtual space, such as flyers, audiovisual informative discourses and the digital press. We selected these discourses because they make up diverse discursive practices in terms of their enunciative, generic, and material characteristics, as well as because they have a large circulation and a wide audience. However, as we will report throughout the article, it is possible to find a certain systematization in the use and appearance of inclusive language that accounts for the dialogical properties that constitute the statements, which transcend the heterogeneity related to the characteristics of the different discursive genres and media. On this occasion, we leave the pedagogical and academic discourses aside, not only because they have already been analyzed in depth in previous works (Tosi, 2019, 2020; Sardi and Tosi, 2021), but also because the objective that guides this study is to investigate non-institutionalized practices that are founded as alternative spaces, prone to linguistic/discursive innovation and that produce different meaning effects. The corpus is made up of 50 discourses, produced between 2018 and 2020, although for this work, due to writing space limitations, we will refer only to some cases, which function in an exemplary manner. Following Courtine (1981), the constitution of the corpus responded, then, to demands of *exhaustiveness*, that is, “not to leave in the shadows any discursive fact that belongs to the corpus, even if it ‘disturbs the researcher”' (Courtine, 1981: 23), and of *representativeness*, that is, “not to extract a general law from a fact ascertained only once” (Courtine, 1981: 23).

### Theoretical Framework

Next, an approach to inclusive language is proposed from the Dialogical Approach to Argumentation and Polyphony (*EDAP* by its initials in Spanish) (García Negroni, 2018, 2019). This approach follows the epistemological assumptions of dialogism (Bajtín, 1982), of the theory of argumentation in language (Anscombre and Ducrot, 1983; Anscombre, 1995; Carel and Ducrot, 2005, among others), of enunciative polyphony (Ducrot, 1984) and of enunciative heterogeneities (Authier-Revuz, 1984 and 1995). As it is known, such theoretical approaches refute some of the most relevant axioms of the formalist linguistic research that was dominant in the 20th century. On the one hand, they question the assumption that the function of language is to represent reality and, therefore, that the meaning of propositions has a truth value. On the other hand, they object to the postulate of the uniqueness of the speaking subject, according to which, per statement, there is only one subject, that is, one individual responsible for everything that is communicated in it.

On this basis, the *EDAP* conceives of statements as answers or anticipations of discourses with respect to which a subjective positioning is always constituted dialogically (Bajtín, 1982). In addition, the *EDAP* views the statement as a response to a framework of preceding shown discourse, which has to be recovered so as to access its meaning; it incorporates dialogic-causal instructions; it analyzes the argumentative chains in a dialogic manner and the dialogic facet in an argumentative manner; it not only assumes a non-unicist take on the subject but also a non-intentionalist and non-voluntary one: in spite of their intension, the subject is not the owner of their own saying; *EDAP* rejects the idea of the enunciator and instead proposes the existence of points of view expressed in the statement and conceives of the speaker, S, responsible for the enunciation, as the trace of the subjective positioning as an answer to other discourses (adherence, irony, criticism, refutation, etc.), captured in the statement. Thus, by understanding the statement as a link in the discursive chain (Bajtín, 1982), *EDAP* analyzes the different subjective positionings that are argumentatively manifested in the discourse as always dialogical responses to the “frameworks of discourse” that are presented as the cause of the enunciation (García Negroni, 2018, 2019).

In this approach, we refer to Authier-Revuz's (1984 and 1995) perspective on heterogeneities. On the basis of Bajtin's works, which have already been cited, Authier-Revuz studies the status of certain enunciative notions that account for discursive or textual linguistic forms that dilute the image of monodic discourse:

Enunciative complexity is in vogue: distancing, degrees of commitment, enunciative unevenness or mismatch, polyphony, splitting or division of the enunciative subject. such a number of notions [.] serve as evidence of linguistic, discursive or textual forms that alter the image of a monodic message (Authier-Revuz, 1984: 1).

According to the author, there are two great enunciative heterogeneities: the constitutive one and the shown one. The former demonstrates that discourse, despite the subject's pretension that they are an autonomous source of meaning, is constituted by other discourses; the latter alters the apparent unicity of discourse by incorporating other voices with explicit or non-explicit signals. Within this latter group, we can distinguish between unmarked forms, where the presence of *the other* appears without explicit marks, such as free indirect speech, irony and imitation^3^, and marked forms, where the presence of *the other* is univocally distinguished by means of certain linguistic resources: inserted in the thread of pre-existing discourses, the “I” delimits the zones of contact that create the illusion of it being the owner of the words. Some examples of this type of marked shown heterogeneity are direct speech, words between quotation marks or in italics, and glosses. In fact, the use of words between quotation marks and special typography (bold and italics), which are recorded in the textbooks of the different periods under analysis, breaks the neutrality, evidences the inherent polyphony and produces different meaning effects.

In this regard, Authier-Revuz (1995) argues that words marked at a graphic level by means of quotation marks or their equivalent, i.e., italics, consist in a procedure that points to the speaker's judgment about their own enunciation (“autonomic modalization”), although, if the gloss is not explicit, the addressee must assign a meaning to such words. Thus, by locating and exhibiting a heterogeneous element, such graphic marks indicate that the speaker distances themself from, and issues a commentary on, them, which may be about adherence, strangeness, controversy, etc.

To give an example, in Tosi (2018), we showed that, in school textbooks, certain quotation marks express the speaker's reservations about the inadequate or unfortunate character of the denomination in terms of its ideological dimension. By means of the term between quotation marks, there emerges the valuation of S, even when there is no revelation of the one who is responsible for the discourse of others, from which the speaker distances themself (cf. 1). This use allows S to issue a warning about the meaning of the term “Asian barbarism.”

(1) The Huns, dark men with bony faces, small eyes and depressed noses -so strange to European types- lived on horses or in carts, dominating villages in the exercise of what some have called “Asian barbarism.” However, seen as a rebellion against the imperial corruption of the Romans, their struggle could be felt as an executioner of a primitive but destructive justice.

Thus, S refutes the meaning, or the scope, of “Asian barbarism.” In fact, the speaker disagrees with the veracity of the semantic content of this nomenclature and proposes a counterargument by means of the inclusion of the adversative connector “however.” In this way, the speaker refutes the premise that the “Huns were barbarians” and puts forward their own point of view by proposing the following premise: “the Huns executed a primitive justice.”

Also in Tosi (2018), we observe that the special typography (bold, italics, and colored typography) passes comments on the highlighted expression. In this case, S alerts the reader-student to the disciplinary terms or concepts considered important, to which attention should be paid (cf. 2).

(2) Although the driving force behind the economy was rural production, **economic and demographic growth** and the **expansion** of **transport, trade, and industry** led to an important urbanization process, regarding which Buenos Aires was its leading exponent.

Taking into account what has been presented so far, the most characteristic resources of inclusive language are addressed below as marks of marked shown heterogeneity (Sardi and Tosi, 2021), evidencing that they are linked to certain discourse/s to which they allude. Thus, the graphic mark *x* or the morpheme *-e* indicate that the speaker distances themself and makes a comment on such marks, and they produce different meaning effects, as the analysis will show.

## Sexist Uses of Spanish

Studies with a gender perspective make up an interdisciplinary field that takes the notion of gender as a central category. Although the topics have been diverse, since its inception, the studies have expressed the need to problematize linguistic uses in relation to feminisms and sex-gender identities.

If we refer to pioneering work, we must mention Judith Butler's in the 1990s, which promoted the idea that language constitutes a determining factor in the construction of gender. For Butler, the subject is constituted as such by entering language norms, therefore, when not included in the dominant forms, the subject is excluded. The author maintains that placing oneself outside the realm of the enunciable endangers the status of a person as a subject. From such an approach, it can be argued that language impacts on social perceptions and, thus, it is possible to operate on it to make women visible and to show an openness to sexual dissidence forms.

Linguistic studies on gender are profuse, and the relationship between grammatical gender and social gender has been thoroughly examined in several languages, including Spanish (Hellinger and Bussmann, 2001–2002–2003; Pauwels, 2003; Alvanoudi, 2015, 2016, 2020). In this sense, non-sexist and inclusive language would be staging the tension between two types of gender: grammatical and sociocultural. As Ramírez Gelbes (2018a) explains, the grammatical gender corresponds to certain classes of words (the noun, the adjective, the pronoun) and in Spanish it can be feminine or masculine. Due to the same duality, when the grammatical gender refers to sexed beings, it places them in a binary category. Sociocultural gender, for its part, refers to the sociocultural category that is related to the identities of the subjects, and that would object not only to linguistic androcentrism, but also to the binarism given by grammar.

With respect to Spanish, since the 1980s, there have been feminist movements that have promoted actions to eradicate sexist uses. Some of these movements worked for the creation of the *Instituto de la Mujer* (Women's Institute) in Spain in 1983 and the publication of the first guides to non-sexist language, which were employed in public administration. From then on, there began to emerge multiple groups that objected to sexism in language and proposed alternative resources. Among them, we can mention the *NOMBRA* group, *No Omitas a las Mujeres, Busca Representaciones Adecuadas* (NAME: Don't Omit Women, Seek Adequate Representations), created in 1994 and linked to the Advisory Commission on Language of the Women's Institute (Spain), which is responsible for much of Spain's academic production on the subject. Among its members are Carmen Alario, Mercedes Bengoechea, Elira Llendó, and Ana Vargas, who support the thesis of women's lack of symbolic presentation in language. In this regard, they state that the use of the generic masculine to refer to the two sexes does not manage to represent them, since it hides or excludes women, to the extent that it is based on an androcentric way of thinking that configures men as reference subjects and women as subsidiary subjects (1995).

At this point, it should be made clear that, according to Spanish grammar, when things are designated, there is no relationship between grammatical gender (feminine and masculine) and sex; for example, “la cuchara” (the spoon) and “la espumadera” (the skimmer) are feminine; “el cuchillo” (the knife) and “el tenedor” (the fork) are masculine; however, as we know, this categorization has nothing to do with extra-grammatical aspects. Now, as for the words that refer to women or men, there is a match between grammatical gender and the sex of the person [“médic**o**, médic**a**” (doctor, physician)*;* “voluntari**o**, voluntari**a**” (volunteer)]. In addition, when reference is made to nouns pointing to animate beings, the masculine designates the class that corresponds to all individuals, without distinguishing between the sexes. For example, in the sentence “A nivel mundial, unos 18 000 voluntarios fueron inoculados con vacunas experimentales dentro del programa de pruebas para luchar contra el Covid-19” (Worldwide, some 18,000 volunteers were inoculated with experimental vaccines within the testing program to combat Covid-19), the phrase “los voluntarios” includes both men and women because the masculine is the unmarked gender, which refers to the member of a binary opposition and can encompass both members. According to Spanish grammar, this makes it unnecessary to mention the marked term, i.e., the feminine one.

In light of this, academic movements that label Spanish as a sexist language claim that the use of the masculine to refer to both sexes not only causes women's lack of symbolic representation in the language and hides or makes invisible the presence of the feminine gender (Alario et al., 1995: 4), but can also produce ambiguities or misunderstandings. For example, in the case we mentioned of “los voluntaries,” it could generate confusion regarding its meaning: are the volunteers only men or does the noun also include women? In this respect, Ramírez Gelbes (2018b, online) argues:

in many contexts the masculine-understood-as-generic is ambiguous. That is, it is not clear whether one is talking only about men or about men and women. However, there is a point that should be considered: in society today, one does not always speak of binary genders, since the masculine that is said to encompass the feminine also admits representatives who, feeling they belong to other genders, are not represented by the said masculine. This matter also appears to be resolved with the use of “e” as a truly generic and neutral form.

Several guides Spanish and Latin American -especially in the 90s and from 2000 onward- have proposed alternative ways to tend toward a non-sexist language. The following ones serve as exemplification:

- Resources that make the feminine gender visible, such as the split or double mention: “Los voluntarios y las voluntarias” (The volunteers) and the use of bars and parentheses: “Los/las médico/as” y “Lo(a)s medico(a)s” (“The doctors”).
- Mechanisms for gender non-visibility. On the one hand, paraphrase and use of pronouns without a gender mark and employment of abstract nouns: “grupo voluntario” (volunteer group), “el voluntariado” (the volunteer), “quienes se ofrecieron” (those who offered themselves), “personas voluntarias” (volunteer people), among others. On the other hand, certain graphic resources, such as the *at* sign, @, (“l@s voluntari@s”), the asterisk (“voluntari^*^s”), and *x* (“lxs voluntarixs”), which are exclusive to written texts.

In this regard, Ramírez Gelbes (2018a, online) states:

About 15 or 20 years ago, “@” emerged to break with the generic distinction. “Alumn@s,” “chic@s,” or “maestr@s” were used. Sometime later, around 2010, “x” was introduced. Then, we saw words written as “todxs,” “compañerxs,” and “afiliadxs.” However, both forms - which are still seen - collide with the barrier of verbalization. The advantage offered by “e,” is that it can be put into practice in oral language.

However, we must take into account that sexism in language is not limited to morphological aspects, but can also manifest itself at the lexical level (for example, the use of “capitana” (woman captain) to refer to the wife *of* the captain's-, apart from the asymmetrical use of address terms). In the public sphere, women were identified by their marital status (“señora” (madam), “señorita” (miss), by their relationship with a man (“señora de” (Mrs) or “mujer de” (X's woman) and with the use of the masculine form or the explicit use of the noun “mujer” for certain professions and titles: “la presidente” (the woman president) and “la gasista mujer” (the woman pipe fitter).

In short, there have been many academic works and style guides that have been produced in Spain since the 1980s, but in Argentina the debate was established a few decades later, as is analyzed in the following section. In fact, in Argentina, non-sexist language began to emerge massively in social discourses thanks to the “Ni una menos” (Not One [Woman] Less) actions, a feminist collective formed in 2015, which opposes violence against women in all its forms. In their discourse, one of the most frequent mechanisms to make women visible and to refer to the feminist struggle is the use of the feminine variant of nouns, pronouns and adjectives. This mechanism affects the evident configuration of a specific gender collective: “Ni **una** menos”; “**Vivas** nos queremos” (We Want Us [Women] Alive), “Si nuestras vidas no valen, produzcan sin **nosotras**” (If our lives are not worth it, produce without us [women]), among many other phrases (Sardi and Tosi, 2021).

### Gender Inclusive Language

Thanks to the struggle of the LGBTTTIQ+^4^ collective, the so-called “inclusive language” was configured as an alternative to account for sexual dissidence forms and to escape from Spanish binary system: feminine-masculine.

Inclusive language proposes resources for the non-visibility of gender - which we mentioned in the previous section, but rejects the use of the feminine variants, since it objects to binary forms. In addition to the graphic resources already addressed -the *at* sign, the asterisk and *x*, there is the *e* morpheme, which began to circulate widely in 2018, with the marches and discourses that supported the *Interrupción Voluntaria del Embarazo* (Voluntary Interruption of Pregnancy) Bill (2018), as we have already mentioned.

According to Martínez (2019), in the case of inclusive language, speakers are proposing a change, which points to the language paradigm, insofar as the use of the *-e* phoneme acquires the status of a morpheme, since it becomes filled with meaning. Thus, the choice of this morpheme is the matrix of a possible reconstruction of the gender paradigm which, as stated by Martínez (2019: 11), is constituted as follows:

MASCULINE: -O (S)
FEMININE: -A (S)
OTHERS: -E (S)

Therefore, “the semantic substance that categorizes the plural paradigm would be: ‘different from one + gender' and the gender type categorized as masculine, feminine, and others” (Martínez, 2019: 12). But as the specialist points out, “the category others would correspond to what is neither masculine nor feminine because, either they are both at the same time or it is a different option” (Martínez, 2019: 12). However, in this description it does not consider, the option that the morpheme “-e” could include not only “both [female and male] at the same time” or “a different option,” but also those two options at the same time: women, men, transgender, transsexual, intersex, lesbian, gay, etc. Considering that Martínez's classification does not take into account the problem of the universal/generic masculine gender, in this article it is proposed that the “-e” could be not only “others” but also “all.”

Let's see, as an example, the following posts on social networks, which circulated in 2020:

(1) Les vecines de Adrogué aplaudimos al personal de salud que atiende a les enfermes contagiades de COVID-19.
  The neighbors of Adrogué applaud the health personnel who take care of COVID-19 infected people (Publication on the Facebook wall of a neighbor of the town *Adrogu*é, located in the province of Buenos Aires, August 23, 2020).
(2) ¡Feliz día a los maestros, las maestras y les maestres!
  Happy Teachers' Day! (Publication on Instagram of a message from a children's literature publisher on September 11, 2020, the day that Teacher's Day is celebrated in Argentina)

Accordin to Martínez, in example (1) the use of the *-e* morpheme is detected as a neutral gender replacing the established binary genders. The morphological changes are applied throughout the phrase, on articles, nouns and adjectives: “Les vecines” and “les enfermes contagiades.”

Here *-e* is neither feminine nor masculine, because it is others, or it makes no difference, according to Martínez. But, in reality, “vecines” and “enfermes” could be saying that they are people with different gender identities, or even that the speaker does not know their gender identities. We really don't know if the “vecines” or “enfermes” are only men and women, or if they are, for example, transgender people and men. With respect to (2), an alternative can be observed in the use of the *-e* morpheme. Although, as we saw in the previous example, *-e* replaces the generic masculine variant, here a splitting into three genders is produced: masculine, feminine, and one that encompasses the gender dissent not included in those two options. That is, the two binary genders are distinguished (“los maestros y las maestras”) and a third one of a dissident type is included, which objects to such binarism (“les maestres”). This last case corresponds to the interpretation of -*e* as a different option from masculine and feminine.

As for the -*e* morpheme, in its beginnings, its use was limited to informal contexts, both in oral and written discourse. However, it is worth pointing out that, in the last year, inclusive language has begun to be legitimized in more formal discursive practices and institutions, such as schools, teacher training courses and universities. Gradually, in Argentina, books are also being published employing inclusive language, corresponding to different discourse genres: literature, cookbooks, journalistic reports, etc.^5^. In the last few years, teacher training institutions and universities have even recognized as valid the use of inclusive language by students in their writing of evaluation productions^6^.

In this work, as we have already mentioned, we analyze texts written in external inscriptions of the urban environment and in certain discourses that circulate in virtual space. In them, besides the *-e* form, we have found uses of *x*, but we did not find any of @ and ^*^, so we propose that the latter be resources falling into disuse.

Language academies, among which is *Real Academia Española* (RAE), object to non-sexist uses of language, due to their ungrammaticality in the case of @, *x*, and *-e*, or because they deem them artificial and unnecessary, regarding the employment of gender-form splitting, paraphrasing or abstract nouns. With respect to RAE's position, Ramírez Gelbes (2018b) states that it is a very conservative institution, whose purpose and function is to maintain the uses and customs of the Spanish language: “It is only logical that it rejects the imposition of a language that creates a third gender that does not exist in Spanish” (Ramírez Gelbes, 2018b, online)^7^.

Without a doubt, there are conflicting aspects around inclusive language that are linked to theoretical and methodological matters, and, at this point, the conceptualization of language that is upheld and the theoretical framework that is adopted are vital. One of the most recurrent objections to inclusive language is that it is consciously put forth and planned by a minority group, usually characterized as educated, middle class and urban. For Moure (2018), it is not “a change ‘from below',” that is, originated as a progressive and generally slow expressive need of a considerable number of speakers, but rather, it is a proposal “from above,” springing from a numerical minority, born of a middle class group that seeks to impose, by means of a mark on the language, a value around a social claim.” Likewise, Company Company (2019) expounds that, by nature, language is inclusive and that speakers are free to use it and no one should force them to speak in a certain way. “Any imposition on how to use language is an authoritarian act” (Company Company, 2019: online). In turn, Escandell Vidal (2019) points out that it is not acceptable for a group to unilaterally arrogate to itself the representation of, for example, “all women” and to feel legitimized to create a division, which she characterizes as “Manichean and exclusive.” Furthermore, she warns about the danger entailed in attacking the language system. In this regard, Lauría and y Zullo (2018): 2) state that the “problem” is that this time the variation is not the result of a spontaneous or unconscious process, but rather a *glotopolitical* intervention on the public use of language, which implies a conscious and deliberate action and, in many cases, an “activist gesture.” The new linguistic and discursive pattern, then, is not provided by the school, the university, the academy, or the mass media, but by certain activisms born on the margins of groups with power over language, such as the feminist and the LGBTTTIQ+ collectives. According to Lauría and y Zullo (2018), language undergoes continuous innovations, such as the introduction of neologisms or the emergence of phonological variants, without causing great controversy or social debate, but inclusive language is different, because it is managed in a planned way and by minority groups that have been historically marginalized.

Another position that objects to inclusive language refers to the arbitrariness of grammar. For example, Company Company (2019) states that grammar does not necessarily reflect the world and its binary organization consists in an arbitrary fact of secular sedimentation and millenary legacies. In contrast, Martínez argues that grammar (morphosyntax) is ideologically and communicatively conditioned and is shaped in accordance with the communicative needs of its speakers. Thus, linguistic change occurs when certain successful communicative uses, accepted by the community, crystallize into grammar. In this way, “The values of a culture -its social biases- are often reflected in language: not simply in what language has produced as lexicon, but simply in what we are talking about and how we are doing so” (Martínez, 2019: 6). At this point, we consider it relevant to state that our research moves away from thinking of inclusive language as a reflection of society, or as a linguistic or grammatical change. As we make it evident in the analysis, we conceive of it as part of the discursive order; in other words, it is a discursive intervention that introduces implicit comments on the enunciation made and produces certain meaning effects.

In addition, several specialists focus on the fact that inclusive language leads to a system-induced complication, which would result in evident failure. In this regard, Moure (2018) explains that such an intervention would affect the linguistic structure itself and this would represent a much more serious interference. Undoubtedly, inclusive language affects the morphological level and can give way to great changes in the syntax of a text, since it produces modifications throughout the phrase.

In another vein, there is a tendency to state that inclusive language implies a linguistic change. In this regard, Ramírez Gelbez points out that it is not possible to talk about a linguistic change, because “to think that inclusive language will be imposed in 3 months seems a fantasy, since if it is really imposed it will be a matter of decades” (Ramírez Gelbes, 2018b). Although Martínez (2018) recognizes that one cannot speak of change -perhaps it is an “embryo” of change (Martínez, 2018), what is relevant is that the debate exists and makes a “social wound” visible. Martínez makes it clear that the linguistic changes do not determine social transformations, but, in general, it happens the other way around: when societies are transformed, those changes impact on language and new forms begin to be used. In relation to this aspect, what this article seeks to do is to show that the interventions enunciated in inclusive language exhibit a patriarchal discourse from which they dissent, while they formulate new representations. What will happen in the future with inclusive language is unknown: it cannot be predicted whether morphological changes will be systematized in language, that is, if, from a phenomenon linked to discourse, it will become cemented as a linguistic phenomenon. It is a possibility, but it will take decades before we know it.

To conclude, it is relevant to highlight Kalinowski (2018, 2019) position, which states that inclusive language is an eminently political discursive-rhetorical phenomenon. On the one hand, it is a public language phenomenon, that is, it is used in an interview, in an advertisement, in a tweet. On the other hand, every time someone decides to use an inclusion formula, they are making a political statement. In this regard, according to the author, it is not possible to restrict people's freedom to make political statements, but trying to impose inclusive language on those who do not decide to use it is equally authoritarian, because it forces people to adopt a position they did not take due to their own conviction and initiative (Ramírez Gelbes, 2018a,b). Therefore, this paper proposes to think of inclusive language as a discourse phenomenon, not a language phenomenon. In other words, inclusive forms can be understood as discursive traces, put into play by a group of speakers, but which are not considered elements that have been systematized and incorporated into the language, as it has happened, for instance, with *voseo* (“vos” instead of “tú,” for example, instead of “tú vienes,” “vos venís”), whose use was fought against for decades by the Argentine State without any success. Let us bear in mind that the Argentine State tried, for decades, to eradicate the use of *voseo* through different regulations in school, radio, and written language in general, etc., but without achieving any success. Thus, we see that, when it comes to a language phenomenon, already systematized, impositions do not work and end up failing. Let us remember that, according to Arnoux (2006), discourse is understood as a social practice and, in this respect, the objective of discourse analysis is to focus on the link with the social universe that is evident in these texts in order to investigate the discursive practices connected to social environments. As we have already mentioned, discourse consists, then, in a space that exhibits the traces of the exercise of language left by subjects in a specific discursive genre, which is understood as a “discursive institution” and which implies verbal features associated with a social practice that, in turn, it defines (Arnoux, 2006). In what follows we investigate the resources of inclusive language, inasmuch as they are traces of the exercise of language in concrete discursive practices.

## Analysis

### Inclusive Language in Urban Inscriptions

The inscriptions on resistant surfaces that can be found in the urban space are classified into *indoor and outdoor inscriptions* (Gándara, 2002). While the *indoor inscriptions* are found in bathrooms, universities, walls and transportation seats, the *outdoor* ones are located on public roads: on walls, on posters on light posts or on advertising posters, on bus stops, on square floors, on the asphalt, among other possibilities, and therefore *outdoor* forms are more visible than *indoor* ones.

If we refer to outdoor inscriptions, these can be iconic (images) or predominantly verbal (text). The latter, which are our object of study, usually have, according to Chiodi (2008), the pretension of acting on the addressee, sometimes with artistic intention and other times, to inform and/or persuade the addressee. To achieve this, they deploy ingenious, playful or poetic legends, often related to social denunciations (Chiodi, 2008).

Below, we analyze a series of inscriptions in public spaces that employ inclusive language resources. As it can be seen, they are all short statements that can be easily read by a passer-by. The six inscriptions, presented as examples, are constituted as affirmative clauses, but with nuances that give rise to different meaning effects. Many of them are anonymous; the empirical author of the texts is not made explicit.

**Figure 1:**
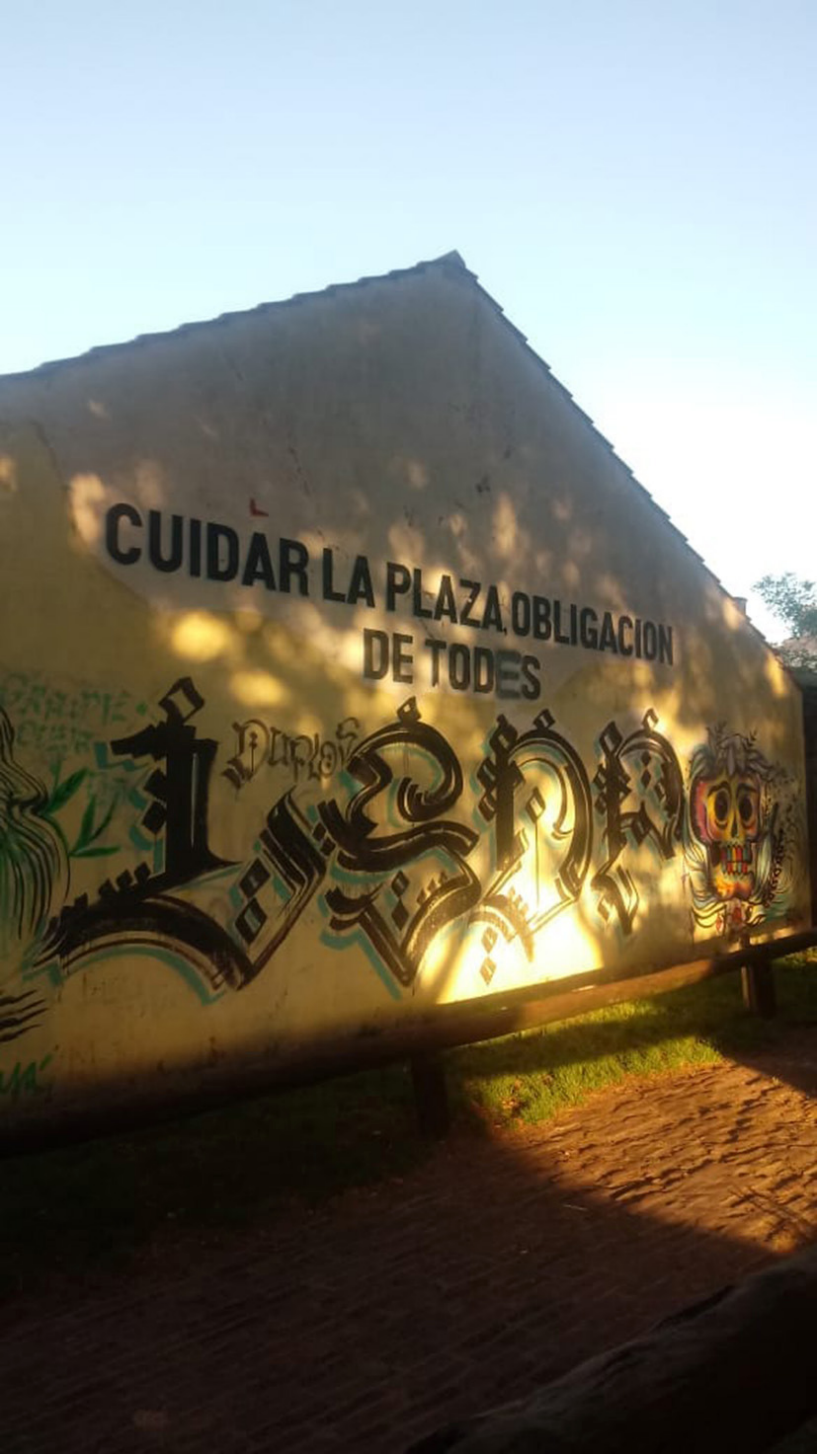
A square in the City of Mar del Plata.

In Figure 1 we can see an inscription, which has been acted upon, in a square in the city of Mar del Plata (Buenos Aires, Argentina) in 2019. As is evident, the word “todos” has been corrected and rewritten as “todes.” That mark clearly shows a discrepancy with respect to the use of the generic masculine form in the original. Thus, two speakers coexist: one who has produced the legend and another who has corrected it and imposed themself on the first one. Hence, it can be argued that the struggle for ways of saying emerges in the enunciation in such inscriptions in the public sphere and dissent is manifested in the discursive materiality.

**Figure 2:**
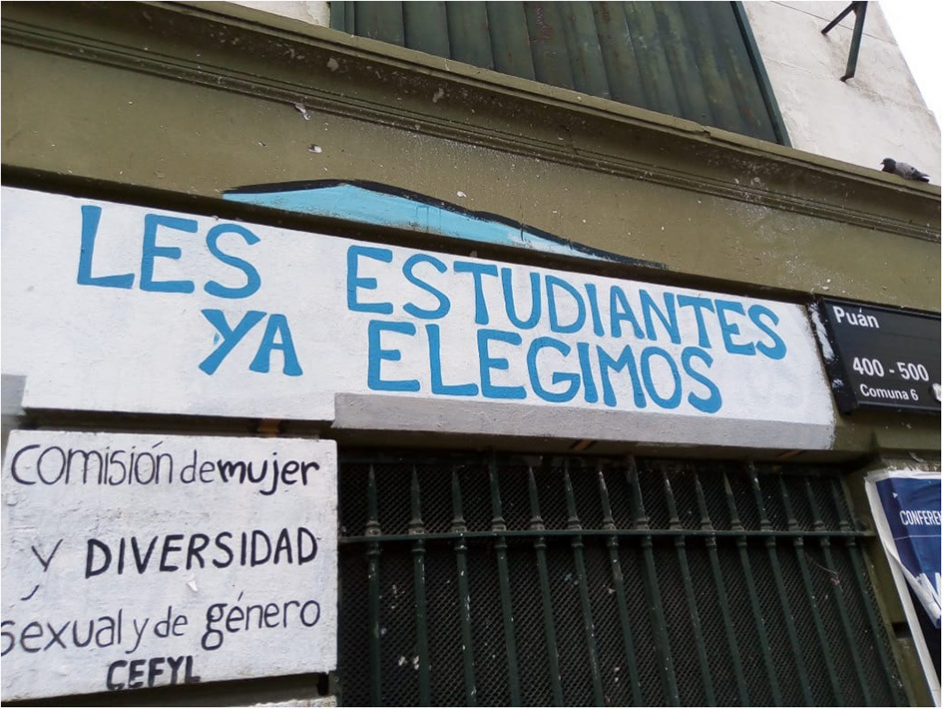
Facade of Facultad de Filosofía y Letras (School of Philosophy and Literature), University of Buenos Aires, City of Buenos Aires.

In turn, Figure 2 shows an inscription on one of the walls of *Facultad de Filosof*í*a y Letras* (School of Philosophy and Literature) of the University of Buenos Aires, in Caballito neighborhood, in the city of Buenos Aires, where it reads: “Les estudiantes ya elegimos.” It is a graffit that was made in reference to gender intersectionality in the political realm of the school. In 2019 (November) by the representatives of the Commission for Women and Sexual and Gender Diversity of the Student Center of the Faculty of Philosophy and Letters (CEFYL).

Although both inscriptions are made up of declarative sentences, Figure 1 conveys a maxim that has an implicit exhortative character. In effect, “Cuidar la plaza es obligación de todes” (Taking care of the square is everyone's obligation) can be interpreted as an appeal to the neighborhood community as a whole: “Todes tenemos la obligación de cuidar la plaza” (We all have an obligation to take care of the square). This is a deontic statement, insofar as it poses the formula “*A* is obligatory,” where *A* replaces a statement that describes an action that is obligatory (taking care of the square). In addition, Figure 2 expresses an assertion made through inclusive “we” and carries the adoption of a stance: not only on carrying out an action (having chosen *X*), but on the construction of a collective S “nosotres les estudiantes” (we, the students), ratified by means of the exclusive plural verb (“elegimos”/we choose). Let us remember that inclusive “we” implies the use of the first person plural which includes the listener (“I, you and possibly” others as opposed to the “I and others, but not you” of exclusive “we”), as is the case that we have just analyzed.

**Figure 3:**
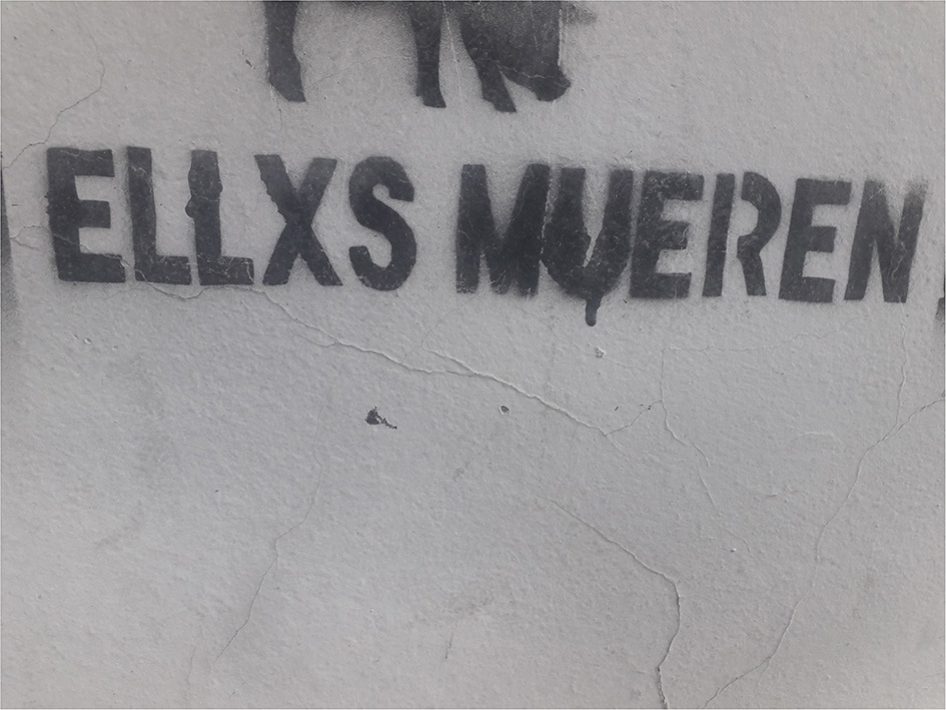
Facade of a house in Lanús, province of Buenos Aires.

Moreover, in Figure 3, we observe the statement “Ellxs mueren,” which refers to an image of low-income boys and girls not meeting basic essential needs, located at the top of the wall. It is a stencil made in the facade of a house in Lanús neighborhood, in the Buenos Aires conurbation^8^. The stencil technique, as explained by Chiodi (2008), consists in making a mold or openwork template, supporting it on a surface and covering it with aerosol. In this way, it can be quickly reproduced on different surfaces. Like all street expressions, stencil requires, in many cases, an addressee who shares the codes that are employed (Chiodi, 2008).

Here, unlike the previous cases, what is displayed is an expression of denunciation of child death due to poverty. In this case, the personal pronoun “ellxs,” which is the object of the enunciation and what is predicated about (“mueren”) is acted upon with *x*.

The three examples show how the “inclusive” act is placed in different elements of the phrase and thus causes different meaning effects. If, in the first case, the relevance was placed on the configuration of the addressees to whom it referred in order to provoke an action involving care and responsibility over the square, in the second, the emphasis is placed on the identity construction of the speaker of the inscription, while, in the third one, the focus is on the object of the enunciation, that is, on what is being predicated. The comments that operate on the introduced inclusive resource can be the following ones:

-*e/x is what is convenient/must be done*
-*e/x with a broad, non-binary value*,
-*e/x in opposition to the sexism of Spanish*,
-*e/x instead of -o*.

Undoubtedly, these comments, raised here in a broad or general way, with the aim of laying the foundations for the analysis, may have a different hierarchy, or they may be different, in each future example according to their meaningful effects.

**Figure 4:**
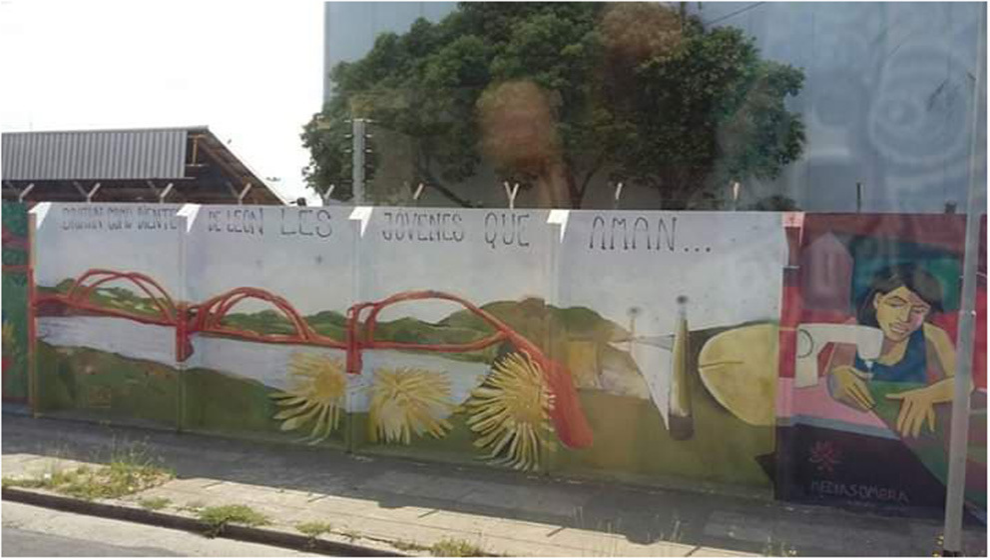
Painting on a wall in Avellaneda, province of Buenos Aires. Photo taken from a bus.

**Figure 5:**
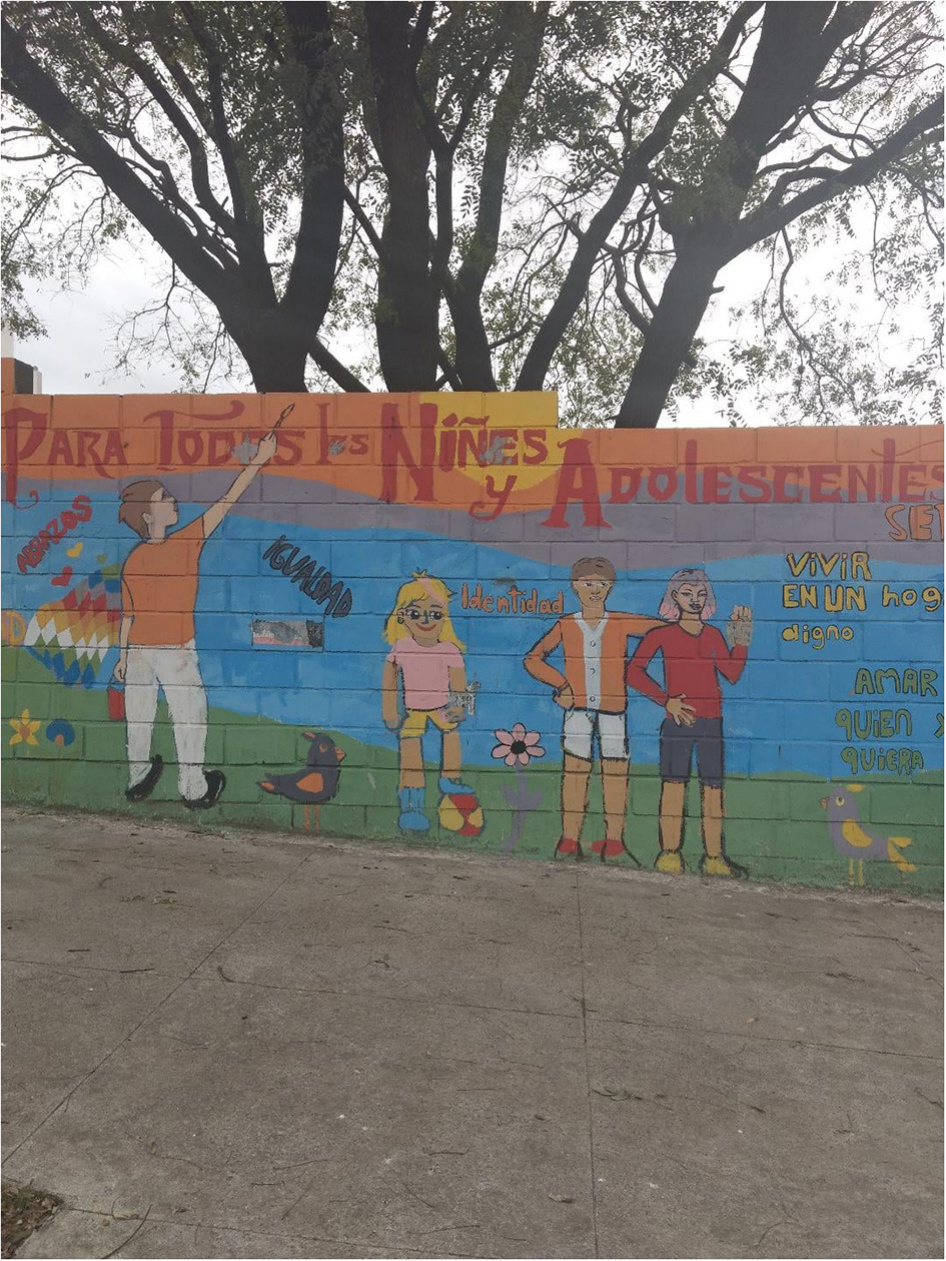
Mural in front of Lezama Park, in San Telmo, Buenos Aires City. Photos: Mercedes Pérez Sabbi.

In contrast, the murals of Figures 4, 5, with poetic tints, are made up of a text and a much larger image. On the one hand, Figure 4 corresponds to a mural on a wall, signed by an artist. It reads: “Crecen como dientes de león les jóvenes que aman” (They grow like dandelions, the young people who love). It is worth mentioning that dandelion is a common herb in Argentina that grows in vacant lots. Without a doubt, it is a statement that vindicates and defends the spirit of youth.

In Figure 5, one can see a very colorful mural, made on Brazil street, in front of Lezama Park, in San Telmo, Buenos Aires City. The mural, which advocates for the defense of equality and identity, is dedicated “PARA TODES LES NIÑES Y ADOLESCENTES” (For all children and adolescents). There, the image plus the nominal phrase in inclusive language (“les niñes”), besides being a clear political and ideological gesture, has a very powerful aesthetic and poetic character that reinforces the gesture of inclusion and respect for diversity. The two examples define the object on which they enunciate from the -e mark. In both cases, glosses 1 to 4 could be applied.

**Figure 6:**
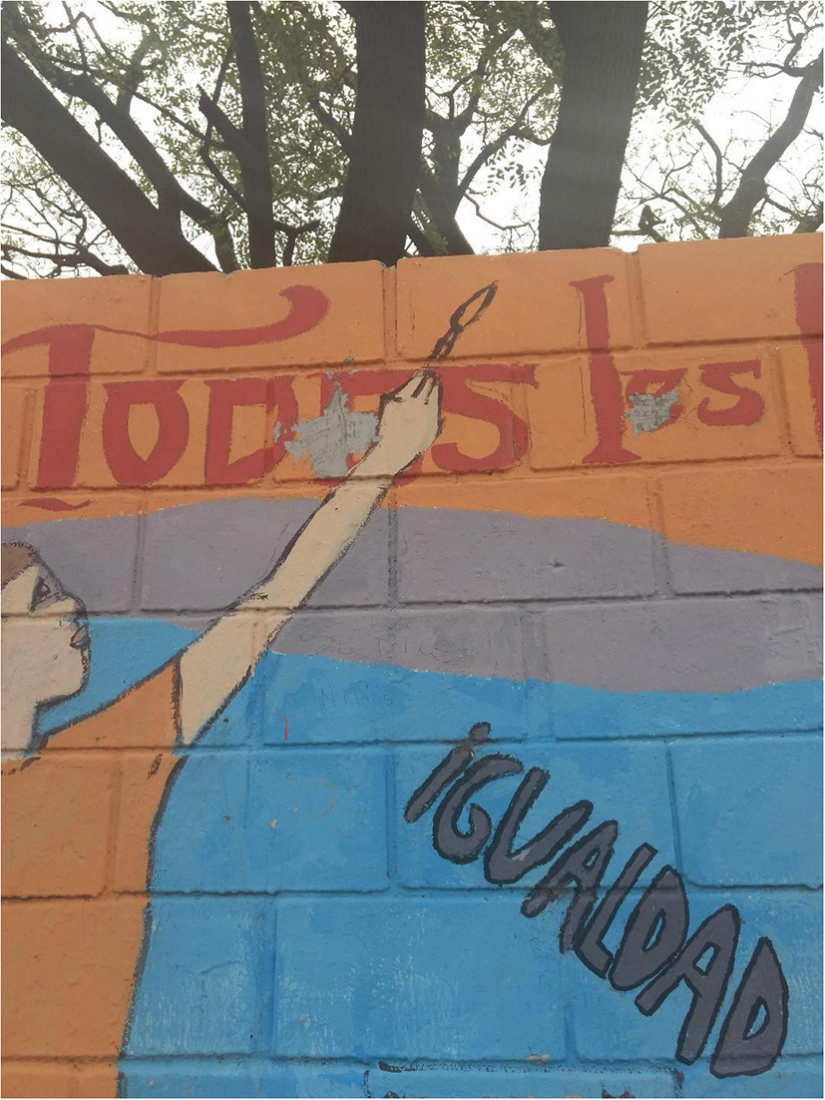
Mural in front of Lezama Park, in San Telmo, Buenos Aires City. Photos: Mercedes Pérez Sabbi.

But if we look in detail at the last mural, i.e., Figure 6, we notice that the *-e* morphemes are acted upon with some gray spots. In this way, we observe a form of intrusion on a public message, which is contrary to what we saw in Figure 1. Here, the spots show the inclusive resources used, conveying a form of repudiation or discrepancy before them.

Considering the theoretical framework established, we see that the outdoor inscriptions that display inclusive language resources can be considered discursive practices that emerge as a space of disruption and interpellation to the passer-by and that interfere in urban space meaning processes. They can also be thought of as spaces of dispute for the meaning and the right to make sexual dissidence forms visible in the public sphere. From such an approach, we think of the city in Orlandi's terms [Orlandi (2001)], as a *symbolic signifying* space, which allows the subject to situate themself in the world because they are located in “the world of significations,” that is, they recognize themself and circulate among the diverse subject positions. Following Zoppi Fontana (1998), regarding her study on urban statements, we can consider these inscriptions as symptoms of the confrontation between different subject positions, from which identification processes are produced, processes which the subjects of social practices in the city constitute. This entails, without a doubt, the emergence of new instances of circulation and legitimization of forms of saying.

### Inclusive Language in the Digital Realm

As it is known, several communicative contexts, which emerged with the advent of new technologies, have given rise to new formats or discursive genres, such as flyers on social networks and informative audiovisual forms, etc. Similarly to what happened in the urban outdoor inscriptions recently analyzed, the texts circulating on social networks appeal to the inclusive trait to configure the different discursive subjects. Some employ *x* to build an identity collective, sustained by the exclusive plural. For example, in (7), S is constructed as part of “trabajadorxs freelance y monotributistas organizdxs” (freelance workers and organized autonomous workers). In turn, “artesanxs, emprendedorxs, productorxs músicxs y maestrxs” (craftspeople, entrepreneurs, music producers and teachers) are the addressees of the other flyers (cf. 8–10), while the configuration of the addressee through the inclusive resources contributes, in addition, to the construction of the image of S (S as publishing house, S as workers' organization, etc.) as more inclusive, open and egalitarian.

(7) Reunión de trabajadorxs freelance. Vamos a discutir sobre los problemas de trabajadorxs freelance e independientes para sumar nuestras demandas a la lucha de Monotributistas Organizadxs. 31/1/2020.
(8) Día Nacional del Músicx. ¡Feliz día a todxs los músicxs! 23/1/2020.
(9) Feria del Faro. Feria colectiva de artesanxs, emprendedorxs y productorxs locales. 15/2/2020.
(10) ¡Feliz día, queridxs maestrxs! Editorial Riderchail. 11/9/2020.

As we have already mentioned, inclusive language generates strong reactions. For example, some of the comments regarding these flyers questioned the use of *x*, some very aggressively. Cases like this set up the discussion about what strategies to take if one wants to convey a message to a wide audience. In relation to the journalistic realm, Ingrid Beck, journalist and director of *Barcelona* (a biweekly Argentine magazine of satirical journalism), asks herself: “When we want to reach other people: what do we do?, do we use inclusive language, which distances us from many?, or do we try to use, for example, neutral words like “las personas,” “la gente” (people)?” (2019). For now, Beck chooses to use some other resources, such as paraphrases or non-gendered pronouns, to resolve such a dilemma, but at the same time trying to reach those audiences who, if they see or hear *e* or *x*, will not read or listen. This is the current trend in mass media: not to use inclusive language, except to provoke some meaning effect, as we see in the following headlines of *Página/12*.

(11) La organización de los nietos de los desaparecidos
  **Les Nietes llegaron para tomar la posta**
  Más de 70 jóvenes de todo el país empezaron a reunirse para “mantener viva la memoria” y también para reconstruir sus propias historias. Enlazan la militancia por los derechos humanos con la lucha por la violencia institucional y por los feminismos y las disidencias sexuales (*Página/12*, August 29 of 2020).
(12) La inteligencia ilegal alcanzó parroquias, comedores y festejos por el día de las infancias.
  **El espionaje macrista no respetó ni a les niñes**
  El Proyecto AMBA de la AFI que conducía Arribas se enfocó, como anticipó Página 12, en control a los partidos políticos y en investigar la actividad de los movimientos sociales en sus locales (*Página/12*, September 27 of 2020).

The headlines in both pieces show inclusive language marks (“les nietes,” “les niñes”), but in the rest of the news text, the generic masculine form is used, as it is possible to observe in the header of (11) (“los nietos de los desaparecidos”/the grandchildren of the disappeared). Why is the *e morpheme* used only in the headline then? Without a doubt, to produce a certain meaning effect and to call the addressee. The use of the -*e morpheme* challenges the reader and calls their attention, besides the fact that its use implies a more “progressive” stance. At the same time, it represents an ideological wink to the target audience of the newspaper, which is usually configured as a “progre” (progressive) reader, open and sensitive to social and gender matters.

To conclude our analysis, in (13) we have transcribed an explanatory video about COVID-19, made by Paula Bombara and Luciano Nieves. It is an informative-educational text for children that has circulated on the networks during 2020.

**“¿‘Qué es el coronavirus?”**

Hola, soy Paula Bombara, soy escritora y bioquímica. Y él es Luciano Nieves, bioquímico y docente de la Universidad de Buenos Aires. Queremos explicarte qué son los virus.

Los virus son parte de la naturaleza pero, pero, los virus no respiran, no están vivos. Por eso no son ni animales, ni plantas, ni bacterias ni hongos. Tampoco están muertos porque tienen la capacidad de reproducirse, claramente, no son como las piedras. No son animales, no son vegetales, no son minerales, son virus. Interesante, ¿‘no?

Básicamente, lo que hacen es reproducirse cuando encuentran materiales para lograrlo. No son ni malos ni buenos. No nos enferman a propósito, tampoco son seres imaginarios, no son monstruos. Los virus no pueden verse a simple vista, se necesita un microscopio especial porque son muy, muy pequeños. Están formados por una molécula de material genético, en la que está la información que necesitan para reproducirse, y proteínas que protegen esa información.

Es gracias a esas sustancias, las proteínas, que pueden entrar a nuestras células. Y es en nuestras células donde encuentran todos los materiales para reproducirse. Para ellos, encontrar una célula, es genial. Pero para nosotres, eso no es una buena noticia. Si sucede en nuestro sistema respiratorio, en nuestros pulmones, como pasa con el coronavirus, lo más común es que entre por la nariz, por la boca o por los ojos. Al principio no vamos a darnos cuenta. Pero luego de unos días comenzaremos a toser, a producir mocos, a estornudar, tener fiebre, temblar. Todas estas son reacciones positivas del cuerpo para sacarnos al virus de encima.

Si nuestro estado de salud antes de la llegada del virus no es bueno, puede que nos provoque alguna complicación mayor. Pero, en la gran, gran mayoría de los casos, después de un tiempo, lograremos superar la infección. Quedate en casa, para cuidarte a vos y cuidarnos entre todes.

*#Quedate en casa*

*Gracias por las ilustraciones a Viviana Bilotti, a Rosario Oliva a y a Eugenia Nobati*.

**“What is the coronavirus?”**

Hi, I'm Paula Bombara, I'm a writer and biochemist. And this is Luciano Nieves, biochemist and professor at the University of Buenos Aires. We want to explain to you what viruses are.

Viruses are part of nature but, but, viruses do not breathe, they are not alive. That's why they are neither animals, nor plants, nor bacteria, nor fungi. Nor are they dead because they have the capacity to reproduce, clearly, they are not like stones. They are not animals, they are not plants, they are not minerals, they are viruses. Interesting, isn't it?

Basically, what they do is reproduce when they find materials to do so. They are neither bad nor good. They don't make us sick on purpose, nor are they imaginary beings, they're not monsters. Viruses cannot be seen with the naked eye, you need a special microscope because they are very, very small. They are formed by a molecule of genetic material, in which there is the information that they need to reproduce, and proteins that protect that information. It is thanks to these substances, the proteins, that they can enter our cells. And it is in our cells where they find all the materials to reproduce. For them, finding a cell is great. But for us (“**nosotres**”), that's not good news. If it happens in our respiratory system, in our lungs, as it happens with the coronavirus, the most common thing is that it enters through the nose, through the mouth or through the eyes. We won't notice it at first. But after a few days we will start coughing, producing mucus, sneezing, having a fever, shaking. These are all positive reactions of the body to get rid of the virus. If our state of health before the arrival of the virus is not good, it may cause us some major complication. But, in the great, great majority of the cases, after some time, we will manage to overcome the infection.

Stay at home, to take care of yourself and each other (“**entre todes**”).

#Stay at home

Thanks for the illustrations to Viviana Bilotti, Rosario Oliva and Eugenia Nobati.

(13) Transcript of the video: “What is the coronavirus?” by Paula Bombara and Luciano Nievez. Available at: https://www.youtube.com/watch?v=lmtWGaa9TPM.

As we can see, the first-person plural pronoun and the indefinite adverb are enunciated with the *-e* morpheme. Thus, “Nosotres” and “todes” refer to an entity that includes the addressee, and is reinforced with other pronominal and verbal marks corresponding to the inclusive we (“nuestros pulmones” (our lungs), “no vamos a darnos cuenta” (we won't notice), “comenzaremos a toser” (we'll start coughing) “cuidarnos” (we'll take care of ourselves etc.). The use of the –e in those cases “agrees and corresponds,” as it refers to a broad and non-binary audience, which can be brown, female or the LGBTTTIQ + collective, an audience that is not always considered in the school environment.

As in the previous cases, inclusive language provokes strong reactions and, on many occasions, there is a great resistance to reading or listening to it. In fact, several school principals decided that the video should not be show in their institutions due to, exclusively, the use of inclusive language.

In these virtual spaces, also, the use of inclusive marks points to a configuration of a certain dialogical positioning, which presents an image of patriarchal discourse that gives way to it and from which it dissents: *-e/x is what is convenient/must be done, -e/x with a broad, non-binary value, -e/x in opposition to the sexism of Spanish, -e/x instead of -o*. The same discourse reveals, thus, a sexist, patriarchal image of the Spanish language, which it comments on and refutes, while at the same time proposing new subject representations.

## Discussion

As we have already pointed out in the previous sections, for various researchers, inclusive language is a proposal that seeks to impose a value around a social claim (Moure, 2018), or a Manichean and exclusive proposal (Escandell Vidal, 2019). For all these reasons, it would be unfeasible and its use, unacceptable.

In contrast to this position, other researchers argue that language accounts for social reality and therefore transformations take place and are necessary. In this respect, Cartín (2010) maintains that language is a reflection of society and that the changes that occur in it respond to the social changes that are gradually unfolding. From this perspective, then, inclusive language can be conceived of as a social transformation mechanism. In turn, Martínez (2019) makes it clear that the changes made in language do not determine social transformations, but that, in general, the opposite is true: when societies are transformed, those changes impact on language and new forms are generated.

In contrast to these positions, and according to the analysis performed, in this work we consider, as do other specialists (Minoldo and Balián, 2018; Glozman, 2019a,b)^9^, that language does not reflect society nor does it have a referential function, that is to say, not because one speaks with “inclusive” features will society be more inclusive, nor the opposite. Hence, we propose that each statement in which inclusive language is used can be understood in dialogical terms since it arises as a response to a previous patriarchal discourse that S refutes and comments on.

Considering this framework, we propose that *x* and -*e*, the characteristic resources of inclusive language, be considered as marks of marked shown heterogeneity (Sardi and Tosi, 2021). From this perspective, the words or expressions used with some of these elements express a commentary by the speaker on their own enunciation, which manifests: (1) dissenting from the generic masculine variant, or rather, from grammatical binarism (feminine-masculine), (2) proposing a new variant that breaks with binarism, and (3) evoking other related discourses that support the speaker's saying (i.e., gender theories and guides to inclusive language). As we have already pointed out, the comments of S (among others) can point to the following: (1) *X/-e is what is convenient/must be done; (2) X/-e in the absence of other options/resources are not present; (3) -o and -a are not valid, thus, X/-e; (4) X/-e instead of -o; (5) X/-e, as gender studies dictate, or as inclusive language guides recommend, among others*.

In each one of the texts analyzed in the Analysis section, it was possible to notice the positions of S in the face of other discourses, as a way of accepting political movements of gender or social demands of gender and inclusive language guides, or as a form of criticism or rejection of generic use of the masculine in Spanish grammar and legitimized by society.

By way of illustration, a note from a thesis written entirely with the *x* mark is added, in which the commentary on the enunciation is made explicit. The use of *x* is explained in the following terms:

The way we conceive of sex and gender is not independent of the way we name them, and therefore, represent them. Given that in this thesis I take up an explicit position against gender binarism (the belief that there are only two sex-gender possibilities, masculine and feminine), in order to reflect that position in my writing I have opted, among other possible forms, for the selective use of the x-ending, understood as the indicator of a diversity that goes beyond or transcends the masculine/feminine binarism. In some cases, the use of a masculine grammatical gender was intentionally maintained, to refer to historically patriarchal actors and institutions (Footnote in a doctoral dissertation)^10^.

Undoubtedly, the characteristics of a doctoral dissertation demand the inclusion of a note that explains the editorial decisions made and the reasons behind departing from the conventions and the legitimized language norms.

Moreover, inclusive language forms point to the construction of a certain discursive *ethos*, that is, in Amossy's (dir.) (1999) terms, the image that S constructs of themself in a text. Let us bear in mind that the *ethos* is inscribed in language, and does not correspond to real individuals that are external to the discursive activity (Amossy, 1999). In other words, the *ethos* is configured in the discourse itself by means of linguistic choices. In this respect, inclusive language resources can contribute to the construction of a more egalitarian, flexible and open *ethos*, with certain variations in line with each particular discourse, as we saw in the cases of the publishing house, the newspapers and the informative video.

The *x* and -*e* can be seen in those cases as discursive interventions that show a certain dialogical positioning of S before a discourse framework alluded to in that enunciation. In effect, they evidence a dispute against the use of the generic masculine form and, therefore, they raise an objection to the discourses considered sexist and patriarchal that circulate and are legitimized in different social realms. Such resources show positioning spaces and the configuration of collectives and of subjects of saying.

Taking into account, then, that the dialogical-polyphonic dimension cuts through the different inclusive language mechanisms, it can be argued that its resources are linguistic marks of dissent, insofar as they function as spaces for the staging of generic otherness and emerge as traces of historically denied diversity. Undoubtedly, all these inclusive language forms generate meaning effects that challenge us as speakers and therefore also make us uncomfortable and destabilize us.

## By Way of Closing

As we have argued throughout this work, inclusive forms are subjectivity marks, which shape discourses that are positioned and differentiated from others. Thus, the words or expressions acted upon with some of the elements of inclusive language (*x, -e*) carry a commentary by the speaker on their own enunciation. The forms of inclusive language point to the image of previous discourses that they present as sexist and patriarchal and with which they disagree. They are forms that object to grammatical binarism, propose new rupture variants and evoke other discourses that have been forbidden up until now. And therein lies the strength and novelty of the so-called “inclusive language”: its marks make room for a questioning, offer new meanings and evoke traditionally silenced voices. Where an *e* or an *x* appears, instead of a masculine mark, there is a commentary that emerges and questions. In line with Zoppi Fontana (1998), we understand inclusive language marks as symptoms of the confrontation between different subject positions, from which identification processes are generated. We hope that the outlined analysis will serve as the basis for future discursive investigations focused on heterogeneities and dialogism.

To conclude, it is worth mentioning that the driving force behind this article has been the desire to contribute to the description of Spanish today and to offer a discursive analysis that puts the focus on the subjective and dialogical dimensions of a linguistic phenomenon enjoying a wide circulation and exerting a great impact in Argentina. We believe that approaching inclusive language can open spaces for debate and discussion. In addition, it can have an influence on the deepening of linguistic reflection and the promotion of respect for diversity from a gender perspective that advocates for a more egalitarian and inclusive society, one which is respectful of differences.

## Data Availability Statement

The original contributions presented in the study are included in the article/supplementary material, further inquiries can be directed to the corresponding author.

## Author Contributions

The author confirms being the sole contributor of this work and has approved it for publication.

## Conflict of Interest

The author declares that the research was conducted in the absence of any commercial or financial relationships that could be construed as a potential conflict of interest.

## Footnotes

^1^Sex implies the physiological and sexual characteristics with which women and men are born. While gender refers to ideas, norms and behaviors that society has established for each sex, and the value and meaning assigned to them.

^2^The inclusive language guides are documents prepared by specific purpose institutions and human rights organisms, with the aim of promoting a non-discriminatory use of language, both in written and oral communications (Tosi, 2020). These are alternative documents to the decisions made by RAE that “in addition to providing style guidelines and recommendations on the use of resources in certain areas (legislative, labor, trade union, etc.), they usually contain sections that make the links between gender and grammar explicit, and explain the use of inclusive language” (Tosi, 2020: 173). For further information on guides to inclusive language, see Tosi (2020); Sardi and Tosi (2021).

^3^*EDAP* analyzes the allusive points of view as traces of unmarked shown heterogeneity (García Negroni, 2019).

^4^With respect to the acronym LGBTTTIQ+, its meaning refers to L, lesbian; G, gay; B, bisexual; T, transgender; T, transsexual; T, transvestite; I, intersexual; Q, queer; and the + sign refers to other identities, such as pansexual, demisexual, asexual, and antrosexual.

^5^To access examples, see Sardi and Tosi (2021).

^6^For example, the Board of Directors of the *Facultad de Ciencias Sociales* (School of Social Sciences) of the University of Buenos Aires decided, through Resolution (CD) N°1558/19, to admit and recognize as valid the use of inclusive language in academic productions at the undergraduate and graduate levels. This measure applies to exams, monographic papers, theses and dissertations. For further information, see Sardi and Tosi (2021).

^7^See Alvanoudi (2015, 2016) and Sardi and Tosi (2021) for a discussion of grammatical gender through the lens of linguistic relativity.

^8^We do not include that drawing due to poor image quality. Unfortunately, the painting was deteriorated and hidden with advertising posters.

^9^We recommend reading Glozman's research (Glozman, 2019a,b).

^10^PhD thesis defended at the *Facultad de Filosof*í*a y Letras* (School of Philosophy and Literature) of the University of Buenos Aires. The title of the tesis was “Los devenires y la identidad de género: hacia un análisis lingüístico-crítico y conceptual de la construcción de representaciones discursivas sobre la identidad de género en Historias de vida de personas trans de la Ciudad de Buenos Aires (2013–2015)” by Soich (2017).
